# Microneedle sensors for dermal interstitial fluid analysis

**DOI:** 10.1007/s44258-024-00028-0

**Published:** 2024-10-01

**Authors:** Gwangmook Kim, Hyunah Ahn, Joshua Chaj Ulloa, Wei Gao

**Affiliations:** Andrew and Peggy Cherng Department of Medical Engineering, Division of Engineering and Applied Science, California Institute of Technology, Pasadena, CA USA

**Keywords:** Microneedles, Biosensors, Interstitial fluid, Wearable

## Abstract

The rapid advancement in personalized healthcare has driven the development of wearable biomedical devices for real-time biomarker monitoring and diagnosis. Traditional invasive blood-based diagnostics are painful and limited to sporadic health snapshots. To address these limitations, microneedle-based sensing platforms have emerged, utilizing interstitial fluid (ISF) as an alternative biofluid for continuous health monitoring in a minimally invasive and painless manner. This review aims to provide a comprehensive overview of microneedle sensor technology, covering microneedle design, fabrication methods, and sensing strategy. Additionally, it explores the integration of monitoring electronics for continuous on-body monitoring. Representative applications of microneedle sensing platforms for both monitoring and therapeutic purposes are introduced, highlighting their potential to revolutionize personalized healthcare. Finally, the review discusses the remaining challenges and future prospects of microneedle technology.

**Graphical Abstract:**

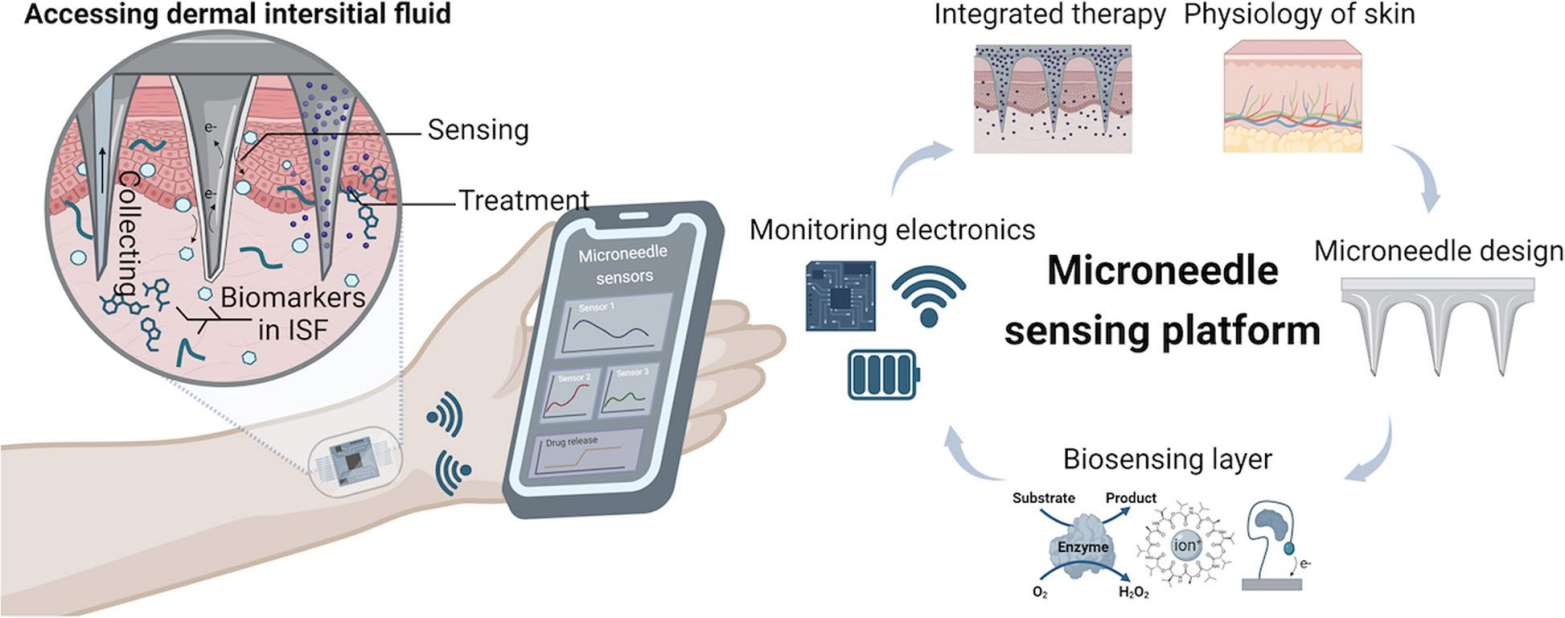

## Highlights

• Microneedle sensors enable minimally invasive and continuous biomarker monitoring through dermal interstitial fluid.

• This review offers a comprehensive overview of microneedle sensing technology.

• Key topics covered include skin physiology, microneedle design, sensing mechanisms, monitoring electronics, and applications.

## Introduction

The ever-increasing demand for personalized healthcare platforms has accelerated the pace of development in wearable biomedical devices for real-time biomarker monitoring and diagnosis. Conventionally, blood-based diagnosis using bench-top analytic equipment has been regarded as the gold standard. However, this method involves invasive and painful blood sampling, often requiring professional medical staff for evaluation. This limitation restricts its usability to occasional snapshots of an individual’s health condition rather than continuous monitoring in daily life. To address these limitations, wearable biomedical sensing devices based on diverse alternative body fluids such as sweat, tears, saliva, and interstitial fluid (ISF) have garnered significant interest [1–5]. Furthermore, advancements in soft and injectable bioelectronics have facilitated the development of continuous biofluid monitoring technologies [6, 7]. These biofluids contain valuable biomarkers indicative of specific diseases and health conditions [8]. Advances in body fluid-based sensing platforms aim to provide alternatives to traditional blood-based diagnostics, offering less invasive, more convenient, and continuous monitoring solutions [9–16].

Microneedle holds promise as a clinical tool to access ISF inside the body in a minimally invasive and pain-free manner. ISF is a biofluid that serves as a medium for nutrient delivery, waste secretion, and molecular signaling between blood and cells, which can be widely accessed from beneath the skin [17]. Since the composition of ISF is derived from blood capillaries, most of the biomarkers found in blood are also present in ISF, which indicates the great potential of ISF as a diagnostic biofluid. To be effective, microneedle platforms aim to reduce the needle’s dimension and penetrate only several hundred micrometers into the skin to access biomarkers in ISF while avoiding potential damage to nerve endings or blood capillaries. Compared to conventional hypodermic needles, microneedle platforms significantly reduce pain, bleeding, and potential inflammatory responses at the application site. This advantage makes microneedles an attractive form factor toward an ISF-based wearable sensing platform.

So far, the clinical utility of ISF has been well demonstrated with commercial continuous glucose monitoring technologies, although they typically use relatively long hypodermic needles [18, 19]. Recently, microneedle-based sensing technology has transcended its traditional role in continuous glucose monitoring to emerge as a versatile sensing platform for monitoring various biomarkers such as metabolites [20–24], ions [25–29], drugs [30–34], and proteins [35–42], coupled with diverse sensing techniques. Moreover, microneedle-based sensors harness miniaturized and integrated monitoring electronics to offer real-time and on-body detection of biomarkers in daily activities [13, 27, 43]. The combination with therapeutic technology can provide a closed-loop healthcare system using a microneedle platform [44–48]. These approaches exemplify significant advancements in the development of microneedle sensing platforms for personalized healthcare.

**Fig. 1:**
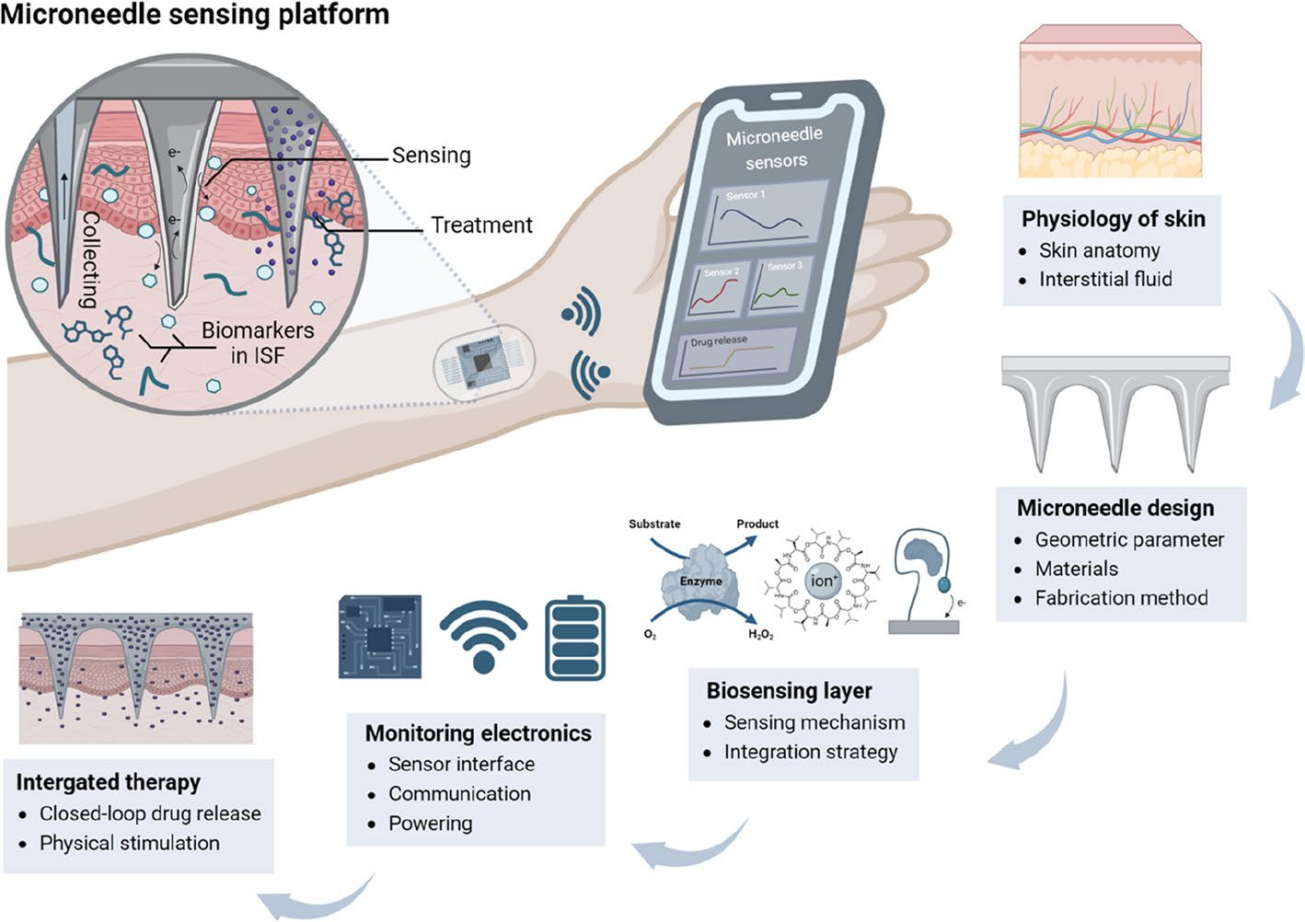
Overview of the microneedle sensing platform. Created with BioRender.com

This review aims to provide an overview of the microneedle sensor technology. The development of microneedle-based sensing platforms involves highly interdisciplinary research spanning from studying the biological aspect of skin to developing integrated wearable modules (Fig. 1). This review explores skin physiology, microneedle design, fabrication methods, sensing mechanisms, monitoring electronics, and applications of microneedle sensing platform. By synthesizing together key findings and technological advancements from the literature, this review seeks to elucidate the potential of microneedle technology in revolutionizing personalized healthcare and advancing the field of wearable biosensing devices.

## Overview of microneedle sensing platform

### Physiology of skin

The skin is a reservoir of ISF, a valuable biofluid source containing many biomarkers that a sensing system may monitor. Microneedle-based sensors aim to create a direct physical pathway into the inner skin layer and extract or directly measure ISF. Thus, understanding skin physiology is critical for designing microneedle structures and sensing elements.

**Fig. 2:**
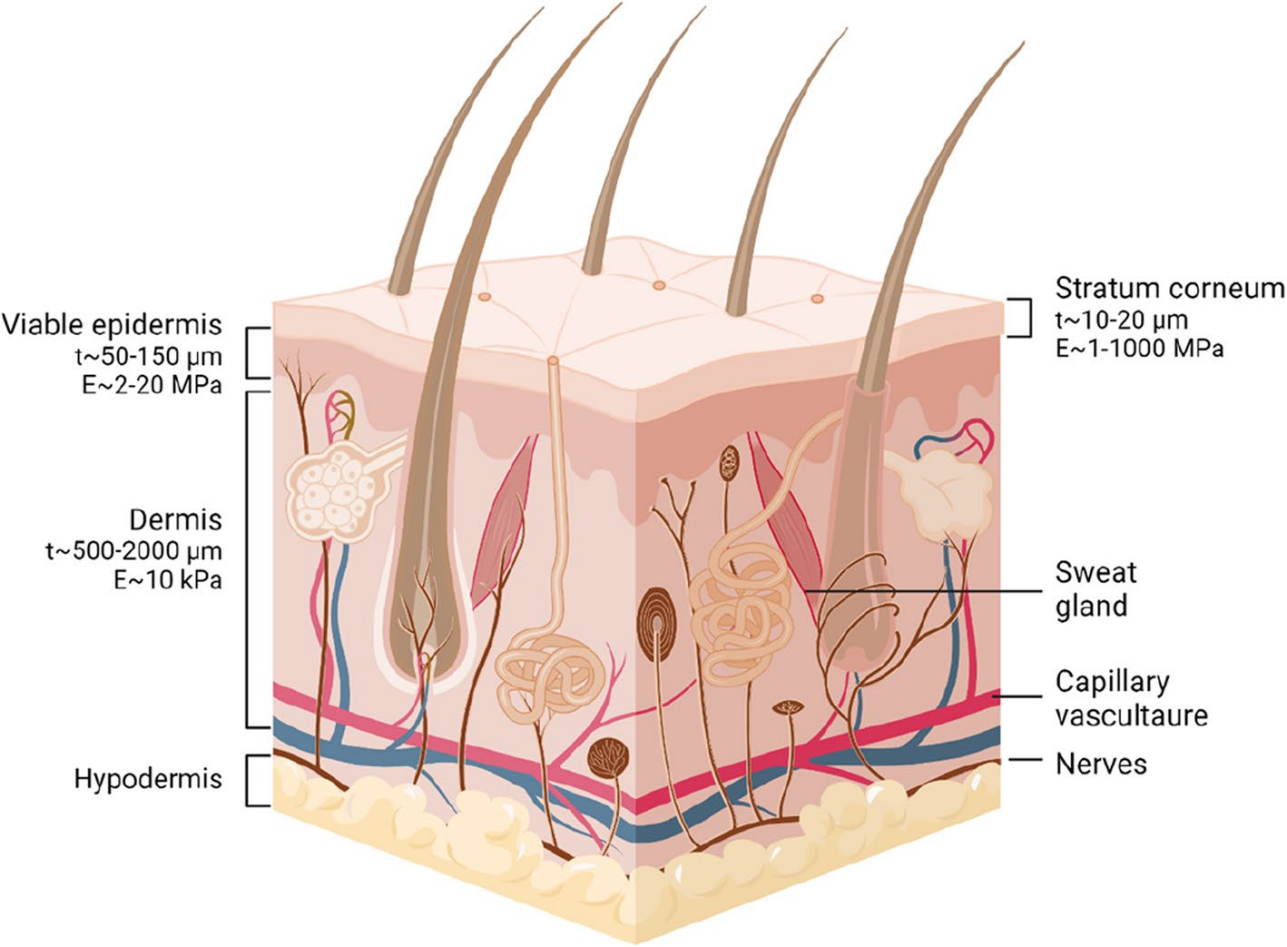
Schematic illustration of the structure of skin layers. Created with BioRender.com

The skin consists of three layers: the epidermis, dermis, and hypodermis (Fig. 2). The epidermis is the outermost and avascular layer of skin. The thickness of the epidermis is generally 50–150 μm and can be much thicker in the palms and soles [49]. It is composed of the stratum corneum and viable epidermis. The stratum corneum is composed of several layers of cells, known as corneocytes, and functions as a physical barrier against the external environment and stimuli such as a microneedle. These layers are also lipophilic and have a higher Young’s modulus (1–1000 MPa [50]) compared to the inner skin layers such as the viable epidermis (2–20 MPa [51]) and the dermis (10 kPa [52]) [53]. Furthermore, the stratum corneum prevents dehydration and protects the inner layers of skin.

The dermis lies underneath the viable epidermis layer. The dermis mainly consists of fibroblasts, collagens, and elastic fibers. Depending on the specific site, the dermis's thickness is between 500 and 2000 μm [49]. Importantly, blood vessels, sensory nerves, dermal lymph nodes, and sweat glands are contained in the dermis. Thus, the anatomy of the dermis must be considered to minimize pain and prevent bleeding. The innermost layer of skin is the hypodermis, with a thickness of up to 3 cm. The hypodermis consists primarily of fat and connective tissues, which store energy and provide insulation to the body.

ISF can be accessible from the skin layers underneath the stratum corneum. Approximately 15–35% of the epidermis and 40% of the dermis by mass is ISF [17, 54, 55]. ISF is derived from blood capillaries [17]. The dermis interfaces with two capillary plexuses, known as the superficial plexus at the junction of the papillary, the reticular dermis, and the lower plexus between the dermis and hypodermis [49]. These capillary interfaces provide many biomolecules an interface to ISF by transcapillary blood exchange. The composition of ISF is similar to that of blood, especially plasma. Many proteins, metabolites, and RNA in blood are also found in ISF [53, 56–58]. This correlation with blood makes ISF a useful biofluid to be monitored. However, it should be taken into account that the biomarkers in ISF may have different steady-state concentrations and time-lags from blood. Depending on the size and hydrophilicity of analytes, the dominant diffusion pathway may differ, influencing the relative concentrations between ISF and blood [17]. Compared to other body fluids such as sweat, tears, and saliva, ISF exhibited low and consistent dilution and high correlation with blood plasma for most analytes [3, 17]. This gives microneedle sensors a distinct advantage over other types of biosensing platforms, such as a skin patch. Additionally, microneedle sensors can access ISF through the skin at a depth of less than 1 mm, providing a less invasive monitoring option compared to implantable sensors.

### Components of microneedle sensing platform

The main functions of a microneedle sensing platform can be divided into three sections: 1) safe and efficient skin penetration, 2) sensitive and selective detection of biomarkers in ISF, and 3) ease-of-use monitoring interface. In addition, therapeutic functions such as drug delivery and physical stimulation have recently been integrated into microneedle sensing platforms. These specific integrations allow for closed-loop treatment toward real-time biomarker monitoring. These specific functions of a microneedle sensing platform should be met to ensure the development of a viable medical device.

Microneedles are created to perforate the stratum corneum and access ISF from the viable epidermis and dermis tissue layers. The geometrical design and material properties of a microneedle determine its piercing capability, pain intensity, and delivery mechanism of ISF to the sensing element (e.g., extracting, absorbing, and directly contacting). The possible design, precision, and available materials of a microneedle are highly dependent on the fabrication method. The fabrication method should be selected considering the desirable microneedle design, manufacturing cost, and time.

Sensing elements selectively respond to specific biomarkers and transduce into a readable output such as electric current or optical changes. Sensing mechanisms with enzymatic reactions, ion-selective membranes, and bioaffinity molecules are already widely adopted for wearable biosensors [1, 16]. These mechanisms can be used as the sensing elements within the microneedle. In addition to the sensor itself, it is necessary to consider how to integrate the sensing elements into the microneedle structure. The integration strategy should deliver enough ISF volume and prevent sensing element degradation over time.

The response of the sensing elements can be measured on-/off-site. While off-site measurement can achieve a simple configuration of the sensing platform, on-site measurement can be portable and incorporate wearable monitoring electronics, which are desirable for real-time measurement of ISF. For electrochemical sensors, monitoring electronics consist of a potentiostat circuit, a power source, and a communicating module. Engineering monitoring electronics is practically important to meet the requirements of overall sensing platforms, such as data precision, communication capability, and power consumption. Moreover, a well-designed wearable monitoring interface can improve overall user convenience.

Real-time monitoring of biomarkers can be useful to provide on-demand treatment. Therapeutic techniques using microneedles have been developed for a long time and can be combined with a microneedle-based sensing platform to achieve feedback-controlled therapy. According to biomarker levels monitored by the sensor, integrated therapy can be activated by a closed-looped system via control electronics. So far, integrated therapy is preliminary, with few therapeutic applications reported. Nevertheless, it is obvious that a combination of microneedle sensing and therapeutic integration is promising.

In the following sections, we will delve into the specifics of each component within the microneedle sensing platform, thoroughly examining their design, functionality, and associated considerations.

## Microneedle design

### Dimension

**Fig. 3:**
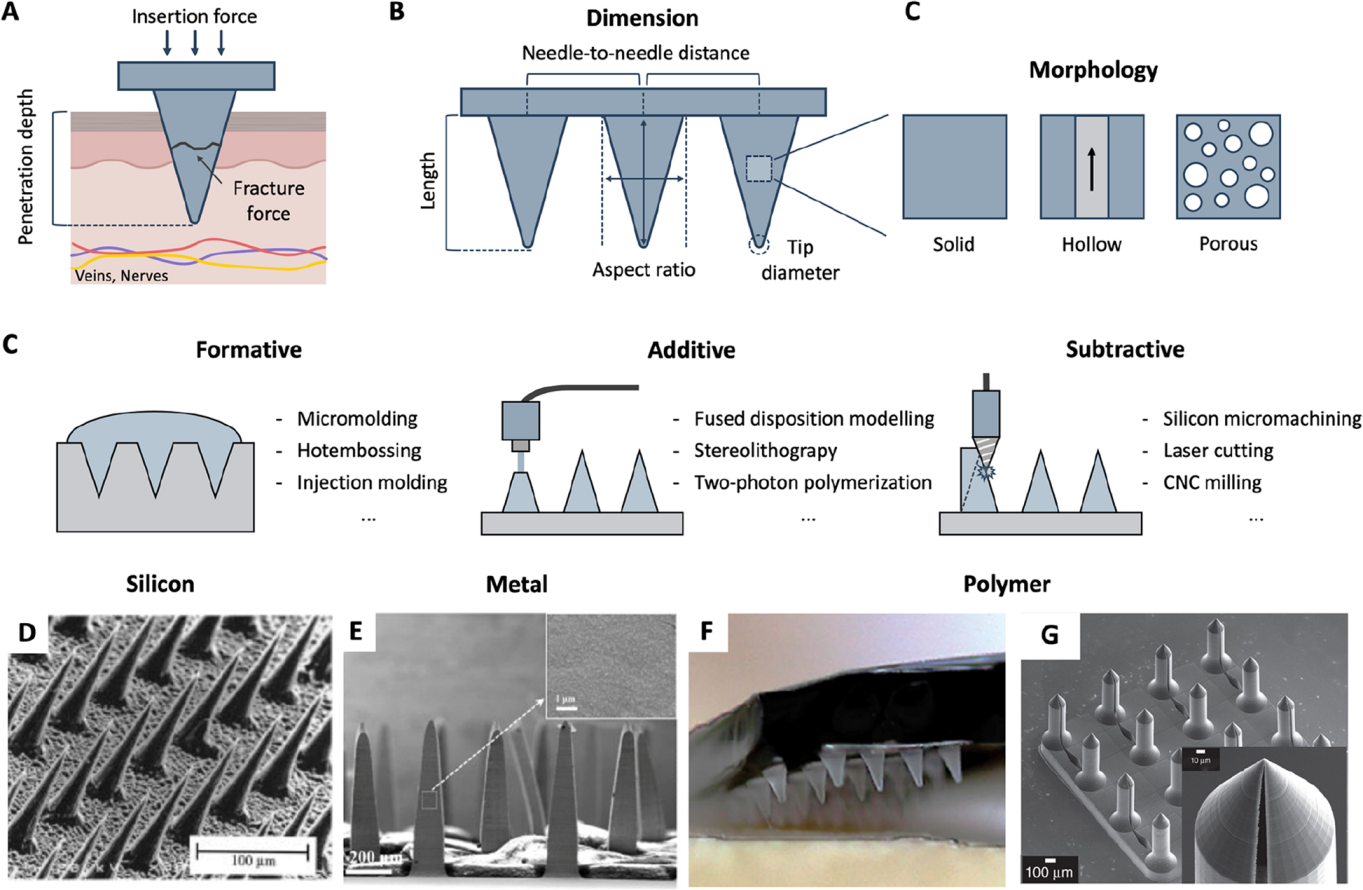
Microneedle design. **A** Considerations for designing microneedle structure. **B** Design parameters of microneedle structure. **C** Types of microneedle fabrication techniques. **D** Scanning electron microscopy (SEM) image of silicon microneedle array fabricated by deep reactive ion etching process. Reproduced from [66] with permission.  Copyright 1998, Elsevier. **E** SEM images of a stainless-steel microneedle array fabricated through laser cutting and bending process. Reproduced from [67] with permission. Copyright 2020, Elsevier. **F** Optical image of a polystyrene microneedle patch fabricated by micromolding. Reproduced from [38] with permission. Copyright 2021, Springer Nature Publishing. **G** SEM images of a two-photon polymerization 3D-printed microneedle array. Reproduced from [68] with permission. Copyright 2017, Springer Nature Publishing. Created with BioRender.com

The microneedle's dimension (e.g., length, tip diameter, aspect ratio, and needle-to-needle space) is related to the mechanical behavior when applied against the skin, such as penetration depth, insertion force, and fracture force (Fig. 3A and B). For the length, the microneedle should be designed to pierce through the stratum corneum (10–20 μm thick [59]) and access the epidermis (50–150 μm thick [49]) and dermis (500–2000 μm [49]). The skin layers have innately elastic behavior. When the microneedle is pushed down to the skin, the skin begins to be compressed first, and with enough applied force, the skin will perforate. For this reason, the actual penetration depth inside the skin is shallower than the length of the microneedle, around 10–80% of the length [60]. Therefore, the length of the microneedle should be long enough to reach the inner skin layer. However, at the same time, it should be considered that a longer microneedle is riskier for disturbing nerve endings and causing pain when using the microneedle. A previous study demonstrated that a 1450 μm-long microneedle needle induced 7.5 times higher pain intensity than a 480 μm-long microneedle [61]. Furthermore, bleeding from the dermal capillary is another health risk of a longer microneedle design. In addition to the length, the tip diameter influences the penetration depth [60]. A sharp tip is more likely to perforate the skin with minimal deformation, thereby enhancing the penetration of the microneedle. Application velocity [62] and applying force [63] are also important factors in penetration depth. In the case of a microneedle array, a larger needle-to-needle distance results in deeper penetration [64, 65]. These factors should be considered together to optimize the length of the microneedle to access ISF from the inner skin layer without pain and bleeding.

Mechanical stability is also a main engineering design consideration when creating microneedles. Insufficient mechanical stability of the microneedles may lead to their breakage and buckling during insertion into the skin. In addition to insertion failure, broken microneedles may leave debris inside the skin, leading to potentially severe clinical complications. To prevent mechanical failure, the microneedle should be strong enough to withstand the applied force during skin penetration. Therefore, the microneedle's smaller insertion and larger fracture forces are crucial for a robust microneedle design. First, the sharpness of the microneedle tip is the main parameter for determining insertion force. A smaller tip diameter makes a narrower skin contact area, which is advantageous when applying the insertion force within a concentrated contact area to facilitate skin penetration [60]. For example, a previous study found that the insertion force of a hollow microneedle decreases from 3.04 to 0.08 N with a decrease in tip diameter from 80 to 30 μm [69]. With regard to the fracture force, the aspect ratio of the microneedle also plays a critical role. A thicker microneedle will have a lower aspect ratio, which is less vulnerable to fracture [70]. In the case of a hollow microneedle, the fracture force increases with a proportional increase in wall thickness [69]. The dimensions of the microneedles should be designed to secure enough mechanical stability with a proper margin of safety.

### Morphology

To meet the demands of improving mechanical performance and embodying functions like extracting and absorbing ISF, the specific morphology types of microneedles must be considered. The morphology of microneedle structures can be classified into solid, hollow, and porous structures (Fig. 3C). Solid microneedles offer the advantage of the highest mechanical strength, a simple fabrication process, and a wide choice of materials. Solid microneedles are usually coated with a biosensing layer on the surface. Robust solid microneedles have been shown to facilitate skin penetration and allow the biosensing layer to detect biomarkers in-situ within ISF. In addition, solid microneedles can be coupled with ISF extracting methods such as suction [71, 72] and reverse iontophoresis [43], and thereby, ISF is delivered to external sensors from the skin pores created by the solid microneedle. A hollow microneedle has a microfluidic channel inside the microneedle. The channel flows ISF into an external sensor via capillary force [73–75] or is filled with sensing components to measure ISF directly [26, 76, 77]. While advantageous, a downside is that the fabrication of a hollow needle is more complicated than that of a solid needle. Clogging issues and weak mechanical strength of hollow structures are a concern of this morphology design. Porous microneedles are characterized by their microstructure architecture incorporating an interconnected network of voids or channels throughout their structural framework. The porous morphology of the microneedle is adopted to absorb ISF through the needle body [78–83] and increase the microneedle's surface area to enhance the biosensor's performance on the microneedle [84–87]. The porous morphology of microneedles can be achieved by various fabrication techniques such as electrochemical anodization [88], porogen leaching [89], and phase separation [90], tailored to the specific materials used [91].

### Materials and fabrication methods

Materials and fabrication processes shape the design of microneedles for real applications. Material properties such as mechanical strength, biocompatibility, and processability are important parameters. In combination with the dimension of the microneedle, a high Young’s modulus and yield strength are essential to penetrate the skin and prevent fracture. Furthermore, cytotoxicity and the inflammatory response within the skin should be investigated to guarantee biocompatibility. In addition, the type of materials affects the fabrication process and determines the maximum resolution, manufacturing cost, and time.

Generally, microneedle fabrication techniques can be classified into three types: formative, subtractive, and additive methods (Fig. 3C) [92]. Formative methods shape bulk materials into the intended microneedle form using pre-defined master molds. This approach can be simple and cost-efficient. However, the precision of the mold-filling process may be limited by surface tension, which can degrade the final quality of the microneedle [93]. Additive methods create microneedles by sequentially adding materials layer by layer. This approach provides high design flexibility and minimizes material waste. However, the range of material options is usually limited. Subtractive methods selectively remove bulk materials to form the microneedle shape through milling and etching. The precision and scalability of this fabrication process vary depending on the specific techniques and materials used. Generally, subtractive methods offer high design flexibility but do require expensive equipment.

With the three categories of fabrication methods, various techniques have been used for manufacturing microneedles. Each fabrication technique is usually dedicated to a set of specific materials. Hence, the materials and fabrication must be taken into consideration together. Table 1 summarizes representative materials and their fabrication methods. Many materials, such as silicon, metal, and polymer, have been utilized for microneedles. Each material has different advantages and disadvantages regarding their specific properties and fabrication process.

**Table 1:** Materials and fabrication methods

Materials	Main fabrication methods	Main advantages	Main disadvantages	Refs
**Silicon** - High stiffness (E ~ 160 GPa [94]) - Brittle	MEMS-based process (Lithography, Dry/Wet etching)	High resolution Controlled geometry	Expensive Cleanroom requirement	[66, 95–98]
**Metal** - High stiffness (E ~ 50–200 GPa [99]) - Ductile	Laser cutting	Fast and precise	Expensive equipment	[28, 67, 100]
	Electroplating	Design flexibility	Unclean and polluting process	[101–103]
**Polymer** - Moderate stiffness (E ~ 2–8 GPa [99]) - Wide material and fabrication options	Molding	Mass-producible	Limited precision	[104, 105]
	3D printing	Design flexibility	Limited material options	[106, 107]
	Two-photon polymerization	High resolution	Time-consuming	[108, 109]
	Lithography	Controlled geometry	Cleanroom requirement	[110, 111]

Silicon microneedles were the first materials adopted for microneedle fabrication [112]. Silicon and its compounds (e.g., Si_3_N_4_) have the advantage of a high Young’s modulus and good biocompatibility [113]. Notably, a well-established silicon micromachining process can sharpen a microneedle with sub-micrometer resolution. The fabrication process of silicon microneedles generally consists of patterning and etching [95, 96]. Photolithography is used to transfer the master pattern to confine the position and size of the microneedle on a silicon wafer. Subsequently, the patterned silicon wafer is exposed to an etching chemical to form a microneedle in a wet and dry manner. The shape of the microneedle is determined by the preferred etching direction of silicon crystal planes in the case of anisotropic etching or can be formed into a cone-shaped microneedle through isotropic etching. Deep reactive ion etching (DRIE) is a common process of fabricating silicon microneedles. Silicon microneedles can achieve a high aspect ratio, resolution, and density of microneedle array (Fig. 3D) [66, 97, 98]. The main drawbacks of silicon microneedles are the time-consuming and high-cost fabrication, which requires complicated cleanroom processes in a well-controlled environment. In addition, silicon's inherent brittleness can cause a microneedle fracture during insertion and leave debris inside the skin.

The metal microneedle is known for its excellent mechanical properties. Compared to brittle silicon, metal possesses a high Young’s modulus and yield strength. Hence, a high aspect ratio of the manufactured microneedle can be achieved with enough mechanical stability. Various metals, including stainless steel, titanium, and nickel, have been used to fabricate microneedles. Conventional hypodermic stainless steel needles can be converted to microneedles by limiting their length [26, 114]. Laser cutting and electroplating are common fabrication techniques for metal microneedles. An array of solid microneedles can be made by cutting a stainless steel plate (Fig. 3E) [28, 67]. Furthermore, computer-aided design software allows the user to design the shape, dimension, and density of a microneedle to achieve the desired performance [100]. Fabrication techniques such as electroplating can be combined with photolithography [101, 115], drawing lithography [102], and 3D printing [103] to form master structures for electroplating. The metal layer is deposited on the various designs of the master structure, which can be removed for a hollow microneedle subsequently. For clinical implementation, the biocompatibility of metal microneedles should be addressed. For example, metal microneedles can cause immune and inflammatory responses inside the skin [92]. Some metals, such as electroplated nickel, are not biocompatible [116] and should be sealed with a biocompatible layer to avoid a possible immune response.

Polymers have been widely used for microneedles because of their biocompatibility, good processability, and low material cost. In addition, due to a variety of types of polymer materials, polymeric microneedles can possess unique functionalities such as biodegradation [117, 118]. In terms of mechanical properties, polymeric microneedles have moderate stiffness with Young’s modulus ranging from 0.7 to 4.5 GPa, which is lower than that of metallic and silicon microneedles. This stiffness can be sufficient to penetrate the skin with a well-engineered microneedle design. Nevertheless, the high aspect ratio of the polymeric microneedles is limited to secure enough mechanical strength. Polymer microneedles have a wide selection of fabrication techniques including micromolding [119], 3D printing [106, 107], lithography [110], and CNC milling [13]. Depending on the fabrication methods, the resolution and productivity of polymeric microneedles largely vary. For example, micromolding can produce a microneedle in a cost-efficient and mass-producible manner that fills prepolymer into a master mold (Fig. 3F). However, its resolution can be limited because prepolymer is hardly injected into a fine feature of master mold despite the aid of vacuuming and centrifuging. On the contrary, two-photon polymerization enables high-resolution 3D printing of microneedles down to several hundred nanometres (Fig. 3G) [68, 109]. However, due to the serial nature of printing, fabrication time can be quite long, potentially several hours, depending on the resolution and size. The specific fabrication process of the polymer microneedles should be selected to meet the user’s demands.

Hydrogels such as methacrylated hyaluronic acid and poly(ethylene glycol) have emerged as promising materials for microneedles. Hydrogels are hydrophilic polymers capable of absorbing significant amounts of water [120–123]. Due to their swelling properties, hydrogel microneedles can extract ISF through the microneedle body. The extracted ISF can then be analyzed using off-microneedle sensors or embedded sensing elements within the hydrogel matrix. When swollen, the hydrogel microneedles become soft and elastic, which enhances mechanical compatibility with the skin and reduces the risk of needle breakage. Typically, hydrogel microneedles are fabricated through a molding process. Given the relatively weak mechanical strength of hydrogels compared to other microneedle materials, the geometry of the hydrogel microneedles must be carefully designed to ensure mechanical stability.

In addition to homogeneous materials, microneedles can be fabricated using composite materials. Composite materials offer enhanced mechanical strength and additional functionalities, such as electrical conductivity, by incorporating embedded components. For instance, the mechanical strength of polymer-based composites can be significantly improved by incorporating nano-/ microparticles and reinforcers that induce hydrogen bond interactions [124]. Conductive components, such as carbon, metal particles, or conductive polymers, can be embedded within the matrix to render the microneedle conductive [104, 125–129]. This conductive composite microneedle allows electrochemical detection without needing an additional metallic layer.

## Sensing strategies

### Sensing mechanisms

**Fig. 4:**
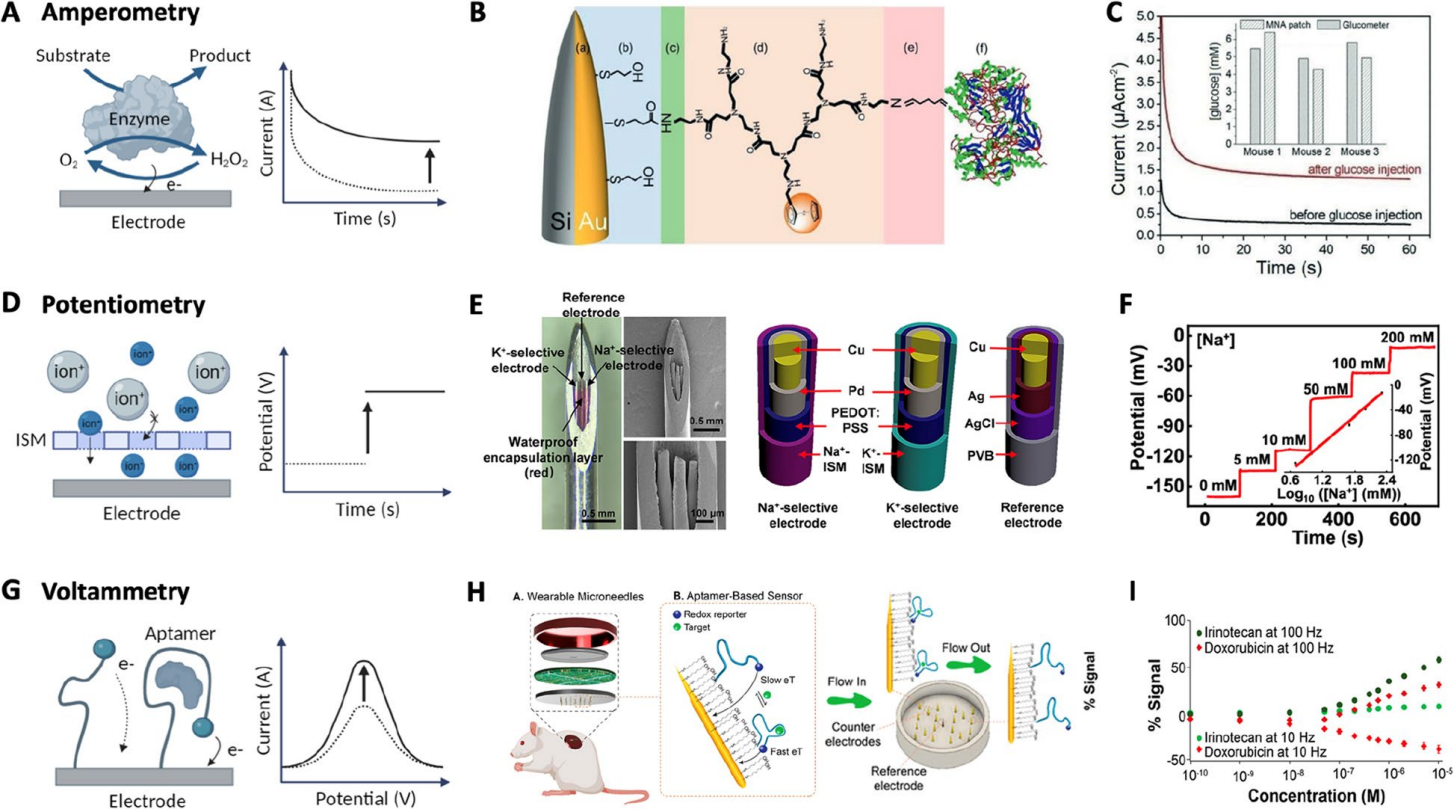
Electrochemical microneedle sensor platforms. **A** Schematic of amperometric enzyme-based sensor mechanism. **B** Glucose oxidase integrated microneedle-based glucose sensor. **C** Amperometric responses of a microneedle-based glucose sensor under glucose injection. Reproduced from [98] with permission.  Copyright 2022, Wiley–VCH. **D** Schematic of potentiometric ion-selective membrane (ISM)-based sensor mechanism. **E** ISM modified microneedle-based Na + and K + sensors. **F** Potentiometric responses of a Na + sensor with increasing Na + concentration. Reproduced from [26] with permission. Copyright 2021, American Chemical Society. **G** Schematic of the voltammetric aptamer-based sensor. **H** aptamer-conjugated multiplex microneedle sensor. **I** Voltammetric responses with increasing irinotecan and doxorubicin concentrations. Reproduced from [33] with permission. Copyright 2022, American Chemical Society. Created with BioRender.com

A microneedle sensor platform could be categorized based on the signal transduction method, such as electrochemistry and optics, as shown in Table 2. Electrochemical sensors transduce an electrical signal—such as current, potential, or impedance—obtained from the working electrode interfacing with ISF to the biomarker concentration in the fluid. These sensors detect signals based on the evaluated electrical parameter through methods such as amperometry, potentiometry, or voltammetry (Fig. 4) [130, 131]. An amperometric sensor measures the current resulting from electrochemical oxidation or reduction of electroactive biomarkers at a constant potential [132–134]. The typical redox reaction utilized comes from an enzyme-catalyzed reaction of the target analytes. For example, an oxidase-catalyzed reaction can generate H_2_O_2_ which can directly react with an electrode or coupled with electron transfer through a mediator layer (e.g. Prussian blue) to an electrode (Fig. 4A) [135]. The enzymatic sensor framework can detect many metabolites and substances [23, 136], such as glucose [98, 137–141], lactate [21, 142–145], and alcohol [13, 146]. A glucose-monitoring silicone microneedle array was developed, where the exposed gold part of the electrode was conjugated with glucose oxidase and dendrimer (Fig. 4B) [98]. The platform inserted into the mouse skin showed amperometric signal changes with an intravenous glucose injection, demonstrating high correlations with blood glucose levels (Fig. 4C).

**Table 2:** Examples of biomarkers and their sensing mechanism

Recognition element	Biomarkers	Detection method	Normal ISF concentration (mM)	Detection range (mM)	In vivo studies	Ref
Enzyme	Glucose	Amperometry	5.2 ± 0.8 [53]	0–22	Human, monkey, dog	[138]
	Lactate	Amperometry	1.12 ± 0.23 [53]	0–30	Human	[21]
	Alcohol	Amperometry	N/A	0–75	Human	[13]
Ion-selective membrane	pH	Potentiometry	pH < 7.35 [53]	pH 3–7	Rat	[147]
	Ca^2+^	Potentiometry	1.183 [53]	0.01–100	Rat	[28]
	K^+^	Potentiometry	3.17 [53]	0–10	N/A	[25]
	Na^+^	Potentiometry	135.7 [53]	0–200	N/A	[26]
Aptamer	Tobramycin	Square wave voltammetry	N/A	0.1–5	Rat	[33]
	Vancomycin	Square wave voltammetry	N/A	0.006–0.042	N/A	[148]
Fluorophore modified receptor	Glucose	Fluorescence	5.2 ± 0.8 [53]	2.8–25	Mice	[118]
	ATP	Fluorescence	4.51 × 10^–6^-3.73 × 10^–4^ [149, 150]	0–3	N/A	[40]
	Cytokines (IL-6)	Fluorescence	9.3 × 10^−9^–2.7 × 10^–7^ [151]	1 × 10^–13^–1 × 10^–7^	Mice	[38]
Chromogenic agent	Uric acid	Colorimetry	N/A	0–1.6	Rabbit	[152]
	Cl^−^	Colorimetry	N/A	20–140	Rabbit	[153]
Plasmonic material	Uric acid	SERS	N/A	0.01–1	N/A	[154]
	Tyrosinase	SERS	N/A	~ 0.05–200	N/A	[155]
	H_2_O_2_	SERS	N/A	0.003–0.1	Mice	[156]

Meanwhile, based on the Nernst equation, the potentiometry-based microneedle sensor measures the potential against a reference electrode to detect ions such as H^+^ [147, 157–159], Ca^2+^ [28], K^+^ [25, 108], and Na^+^ [26]. This potential change can be facilitated by incorporating an ion-selective membrane (ISM), which is a semi-permeable membrane capable of selective reaction with the target ions. As the ions transport across the ISM, the potential difference can be created and transduced on the electrode (Fig. 4D). A multiplex electrolyte monitoring microneedle capable of detecting Na^+^ as well K^+^ via an ISM was developed and displayed log-linear potentiometric responses with an increasing ion concentration (Fig. 4E and F). Furthermore, a voltammetric microneedle system was also reported to detect biomarkers through current measurement over a potential sweep scan. An aptamer, constructed from single-stranded DNA or RNA, serves as the bioreceptor on the voltammetric needle system and undergoes a conformational change upon binding to the target molecule [33, 34, 148, 160]. The structural switching of the aptamer alters the electron transfer rate via a site-specific redox tag, which leads to a distinct signal change (Fig. 4G) [161]. The therapeutic agent monitoring microneedle array was reported to have continuous and multiplex monitoring of the drugs tobramycin, irinotecan, and doxorubicin, as well as its metabolite (SN38) levels. The aptamer conjugated on a gold needle array switches its conformation, interfacing with analytes and improving the electron transfer rate. This results in a current increase proportional to an analyte concentration increase (Fig. 4H and I) [33]. In particular, the microneedle enables continuous in vivo ISF monitoring of tobramycin. Overall, electrochemical microneedle sensors offer low cost, miniaturization, ease of operation and integration, and long-term monitoring [135, 162].

**Fig. 5:**
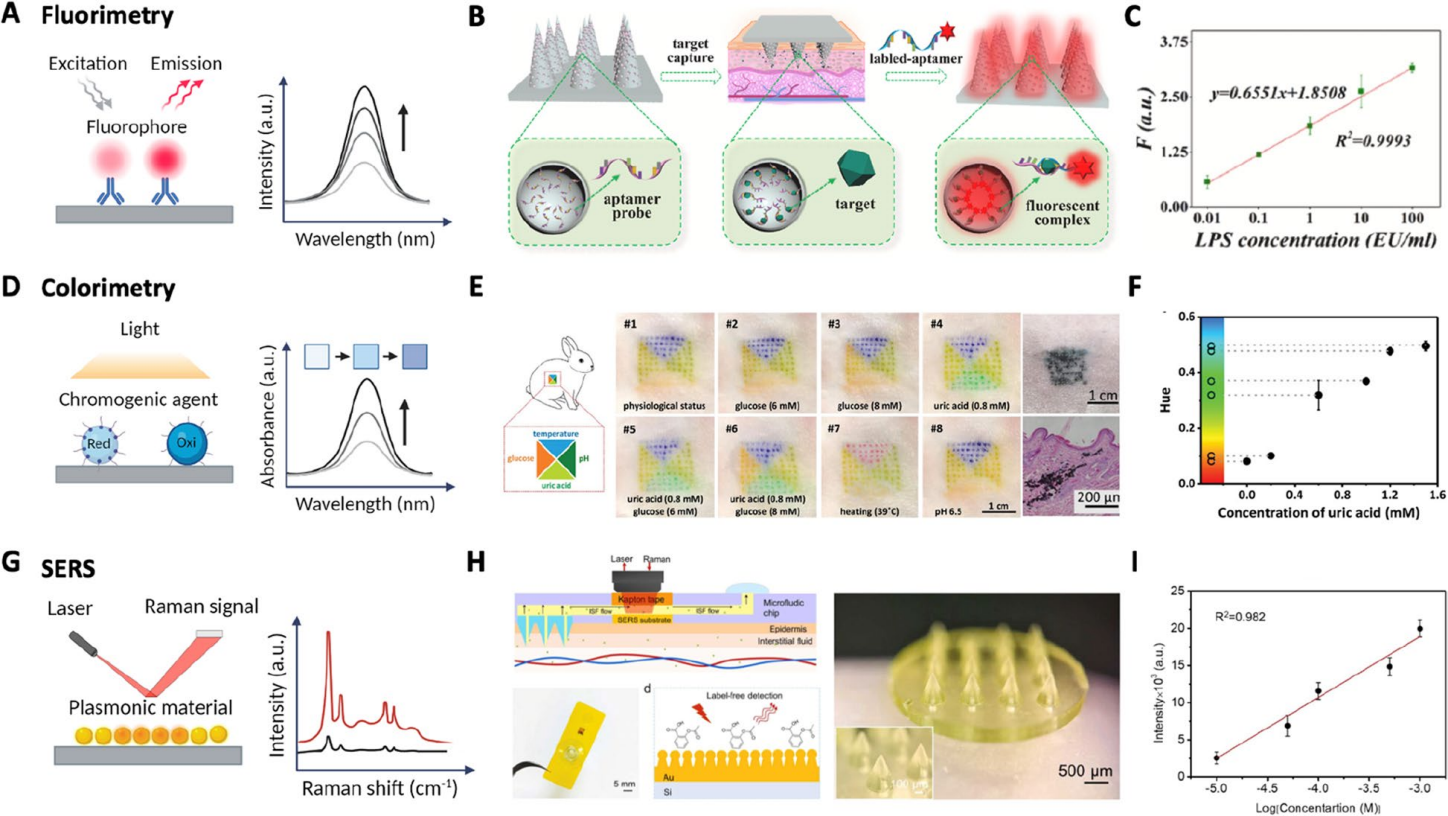
Optical microneedle sensor platforms. **A** Schematic of the fluorescent sensor mechanism. **B** Fluorophore-modified microneedle-based endotoxin sensor. **C** Fluorescence responses with increasing LPS concentration. Reproduced from [164] with permission.  Copyright 2021, Elsevier. **D** Schematic of the colorimetric sensor mechanism. **E** Chromogenic agent-modified microneedle-based multiplex sensor. **F** Colorimetric responses with increasing uric acid concentration. Reproduced from [152] with permission. Copyright 2021, Wiley–VCH. **G** Schematic of the plasmonic sensor mechanism.  **H** Plasmonic microneedle-based uric acid sensor. **I** Surface-enhanced Raman spectroscopy (SERS) responses with increasing uric acid concentration. Reproduced from [154] with permission. Copyright 2024, Elsevier. Created with BioRender.com

An optical microneedle sensor platform includes fluorescence and colorimetric-based methods for quantifying biochemical signals. For a fluorescence microneedle sensor, the fluorophore-modified recognition elements integrated into the electrode emit fluorescent light upon interacting with the target biomolecules [38, 118, 123, 163–165]. Specifically, the excited molecule under light exposure at a specific wavelength absorbs this energy and, once absorbed, emits light with a longer wavelength. This fluorescence change upon biomolecule recognition has allowed quantification of analytes such as glucose, ATP, and small molecules (Fig. 5A) [166, 167]. The microneedle arrays coated with fluorophore-modified aptamer realized highly sensitive and rapid detection of ISF endotoxin with a limit of detection of 0.0064 EU/ml (Fig. 5B and C) [164]. The experimental patch with endotoxin analytes showed more intense fluorescence, leading to a log-linear response from 0.01 EU/ml to 100 EU/ml of endotoxin.

Meanwhile, a colorimetric sensor quantifies the signal through color changes of a chromogenic agent by a spectrometer or with the naked eye. Therefore, the colorimetric system enables easy fabrication at a low cost and serves a wide range of point-of-care diagnostic applications [153, 168]. The chromogenic agents, especially redox-responsive agents, can change their color by inducing the reduction and oxidation states of the agents (Fig. 5D). A multiplex microneedle patch-induced skin tattoo sensor was realized on rabbit skin by encapsulating colorimetric reagents in a hyaluronic acid-based microneedle, which enables pH, glucose, uric acid, and temperature measurements (Fig. 5E) [152]. The changes in each biomarker can be specifically detected through Hue value extraction from the Hue Saturation Value (HSV) color space of the image. However, the color changes are also visible via the naked eye (Fig. 5F). In addition, a surface-enhanced Raman scattering (SERS) based sensor enables highly sensitive and quantitative detection of biomolecules by enhancing the Raman signals absorbed on plasmonic materials such as Au or Ag nanoparticles or metal surface [41, 154–156, 169–171]. The enhancement occurs via the localized surface plasmon resonances (LSPR) effect that amplifies the electric field near the metal surface, and the chemical mechanism (Fig. 5G). A microneedle device was able to integrate a gold plasmonic substrate with a microfluid chip able to sample and deliver ISF from the skin to detect uric acid (Fig. 5H) [154]. Utilizing the advantage of SERS technology, this microneedle system achieved ultrasensitive monitoring of uric acid with a limit of detection of 0.51 µM (Fig. 5I). Overall, optical-based microneedle systems enable a wide range of biochemical detection without electrical stimulation with the analyte, high sensitivity to specific molecules, and rapid response time.

### Integration with microneedle

Integrating sensors with microneedle structures is crucial for enabling on-site, continuous monitoring of biomarkers using a microneedle platform. The integration strategy must ensure adequate ISF transport from the microneedle structure to the sensor. While approximately 120 μL/cm^2^ of ISF is accessible from the dermal layer, microneedle-based extraction techniques can collect a few microliters of ISF within minutes in practical applications [17, 172]. The limited flow rate of ISF can lead to inaccuracies in sensing and delay biomarker detection. Furthermore, the integration method can impact the sensor's stability against mechanical damage during insertion and biofouling within the skin. Considering these factors, the microneedle structures and sensors developed should be properly integrated for reliable sensing performance. The specific integration strategies vary depending on the type of microneedle and sensor employed. An effective pathway and medium between the microneedle and sensor should be considered when designing an integration strategy. The sensing elements can either be placed near the microneedle (off-microneedle integration) or fabricated directly on the microneedle itself (on-microneedle integration).

**Fig. 6:**
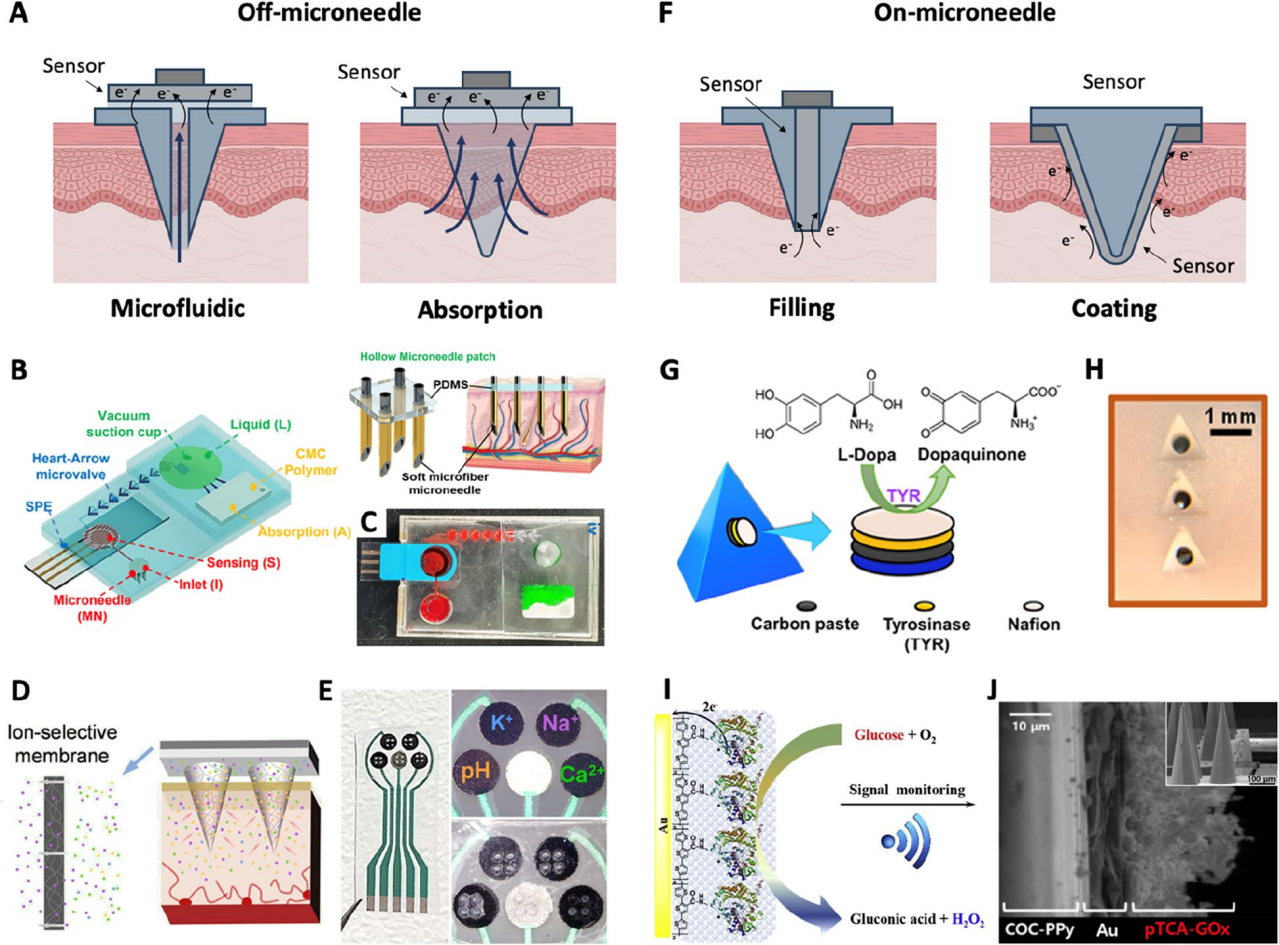
Integration strategies. **A** Off-microneedle integration methods. **B** Hollow microneedle coupled with a suction-assisted microfluidic sensing system. **C** Photograph of the sensing device. Reproduced from [174] with permission.  Copyright 2023, Elsevier. **D** Ion-selective electrochemical sensor integrated with a swellable polymeric microneedle array. **E** Photograph of ion-selective sensor and microneedle array. Reproduced from [177] with permission. Copyright 2023, American Chemical Society. **F** On-microneedle integration methods. **G** Microneedle-based levodopa (L-Dopa) sensor using tyrosinase-modified paste. **H** Photograph of a hollow microneedle filled with the tyrosinase-modified paste. Reproduced from [32] with permission. Copyright 2019, American Chemical Society. **I** Microneedle-based glucose sensor coated with gold and polyterthiophene carboxylic acid -glucose oxidase (pTCA-GOx) layers. **J** SEM images of Au/pTCA-GOx layers on a microneedle. The inset shows Au-coated microneedles before pTCA-GOx deposition. Reprinted from [180] with permission. Copyright 2019, Elsevier. Created with BioRender.com

First, in off-microneedle integration methods, microfluidic channels or absorbing media inside the microneedles are utilized to extract ISF from the skin and transport it to sensors located outside of the microneedle structure (Fig. 6A). This approach allows for separate fabrication of the sensor and microneedle structure, enhancing reproducible sensor performance within the sensing area and sensitivity. In addition, because the sensor is not inserted inside the skin, the sensor is less prone to cause biofouling and physical damage on the sensor. For ISF extraction, a hollow microneedle forms an open microfluidic channel [20, 154, 173–175]. The ISF flows through this channel, driven by capillary force. For example, the hollow microneedles are integrated with an external electrochemical sensor array for real-time on-body monitoring of ketone and glucose in ISF [20]. A suction system can enhance extraction to increase flow rates [154, 173–175]. A soft microfiber microneedle was integrated with a vacuum suction cup, enabling negative pressure-driven traveling of ISF for glucose sensing (Fig. 6B and C). Alternatively, hydrogel microneedles have been utilized to absorb ISF [176–179]. Hydrogels are a type of polymer known for absorbing large amounts of water. When hydrogel microneedles are inserted in the skin, they begin to swell as they absorb ISF. Through the hydrogel matrix, biomarkers present in the ISF are delivered to the sensors located behind the microneedle. Furthermore, since hydrogel becomes soft after swelling, the hydrogel needles have good biocompatibility with the skin. As an example of a hydrogel-based absorbing integration, an electrode array modified with an ISM was coupled with a hydrogel microneedle array (Fig. 6D and E). The ion-selective electrode array was capable of multiplexed electrolyte monitoring in extracted ISF via the hydrogel microneedle.

In the on-microneedle integration strategy, sensors are fabricated directly onto the microneedle itself. On-microneedle integration will allow the sensors to interact with ISF inside the skin, thus eliminating the need for a separate ISF transport pathway. Depending on the sensor location, this approach can be categorized into filling and coating methods (Fig. 5F).

The filling method involves embedding miniaturized sensing elements, such as sensing electrodes wire [105, 146, 181] and enzyme-modified pastes [32, 182, 183], into the hollow cavity of the microneedle. These sensing elements are then able to access ISF through the openings within the microneedle structure. In this architecture, a microneedle structure covers the sensing elements, thereby preventing potential damage to the sensing elements that may be caused during insertion. In addition, the reproducible sensing area of the electrode is the advantage of the filling method. For example, a miniaturized electrode array was fabricated using silicon micromachining methods [184]. The sensing electrode array was modified with glucose oxidase and inserted in a silicon hollow microneedle for glucose detection in ISF. Tyrosinase-modified carbon paste filled in the cylindrical hole of the pyramid microneedle structure (Fig. 6G and F) [32]. The enzymatic reaction of the paste enabled continuous electrochemical monitoring of levodopa.

The coating method, another on-microneedle integration strategy, utilizes a solid microneedle structure as the sensing electrode layer [185]. For modifying the sensing layer on a microneedle, metal layers such as Pt and Au are deposited on the solid microneedle, and then the sensing layer is formed using various coating techniques such as electrodeposition [186–188], dip-coating [113, 189] and drop-casting [31, 145]. This approach allows a straightforward architecture of creating the microneedle sensor and relatively simple fabrication of the solid microneedle compared to the filling method, which requires fabricating a hollow structure. For example, a polyterthiophene carboxylic acid–glucose oxidase (pTCA-GOx) sensing layer was coated onto a cyclic olefin copolymer and polypyrrole (COC-PPy) microneedle by electropolymerization, enabling continuous on-microneedle glucose detection (Fig. 6I and J) [180].

An antifouling layer can be applied to the sensing layer for both off- and on-microneedle integration strategies. The ISF contains various biomolecules, such as proteins and lipids, that can cause nonspecific adsorption and interference. Incorporating an antifouling layer is essential for maintaining long-term sensor performance and enhancing sensor selectivity. Various polymer coatings, such as Nafion [32], chitosan [48], and polyvinyl chloride [13], have been utilized in microneedle sensors. These outer layers can also serve as diffusion-limiting barriers to extend the detection range and act as protective layers to prevent mechanical damage to the sensing layer in the case of on-microneedle integration. Hydrogels such as polyethylene glycol and poly(hydroxyethyl methacrylate) can be adopted to mitigate biofouling and foreign body response [19, 190].

### Monitoring electronics

**Fig. 7:**
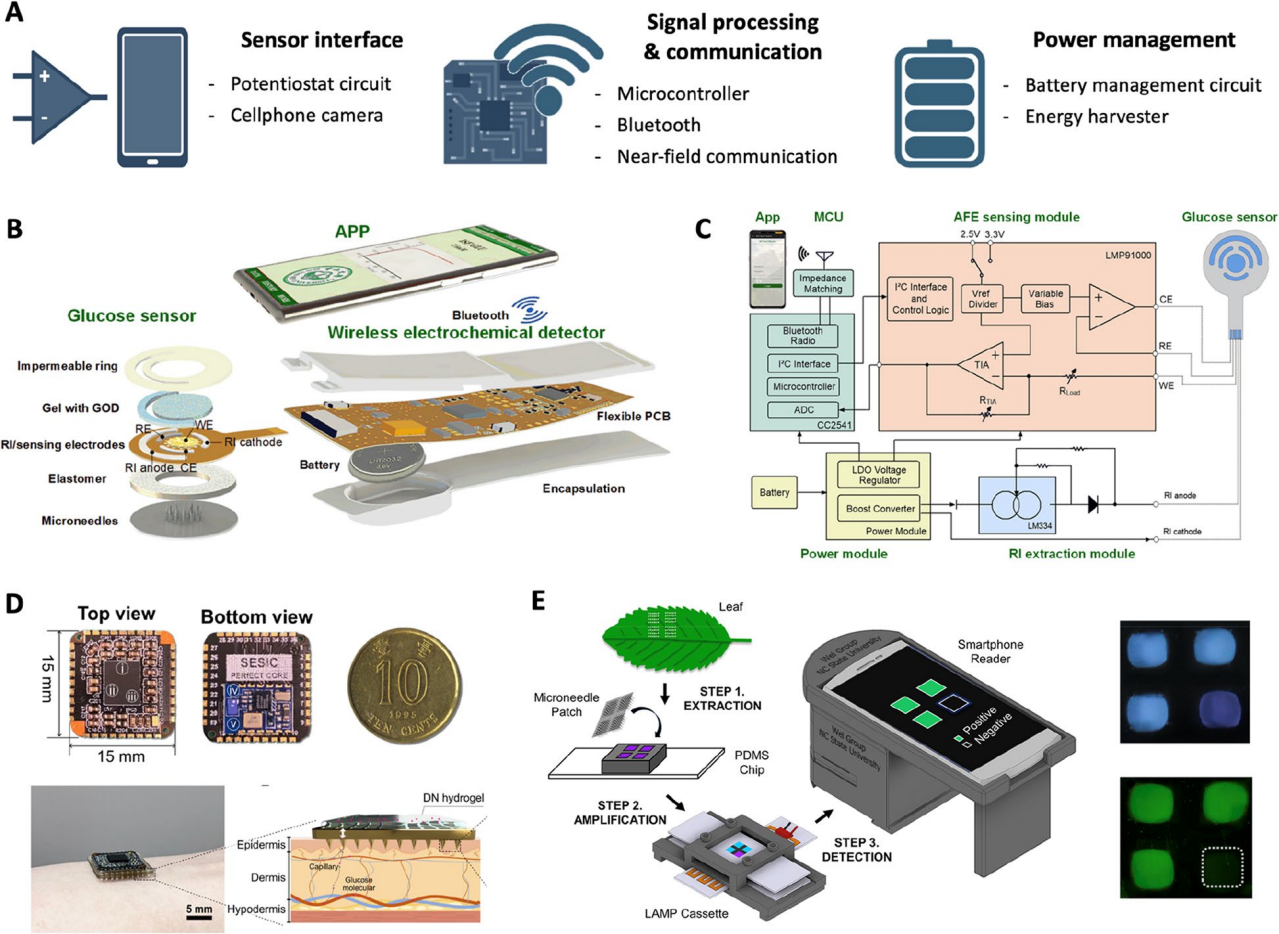
Monitoring electronics. **A** Main functions of monitoring electronics. **B** Smartphone-based integrated microneedle sensing platform for electrochemical glucose detection. **C** Schematic diagram of the smartphone-based integrated platform. Reproduced from [43] with permission.  Copyright 2022, Elsevier. **D** Coin-sized continuous glucose monitoring system. Reproduced from [179] with permission. Copyright 2024, American Association for the Advancement of Science. **E** Integrated microneedle (MN)-smartphone nucleic acid amplification platform. Reproduced from [191] with permission. Copyright 2021, Elsevier. Created with BioRender.com.

System-level integration between microneedle sensors and monitoring electronics is essential for real-time and on-body health monitoring. The main components of monitoring electronics include the sensor interface, signal processor, wireless communication module, and power management circuits (Fig. 7A). Each component should be carefully designed to meet the needs of the microneedle sensing platform, such as the type of transduction method, data precision, wireless communication distance, and power consumption.

The sensor interface is designed to execute various measurement techniques and provide quantitative readings from the microneedle sensors. In electrochemical-based sensing, methods such as amperometry, potentiometry, and voltammetry are utilized to measure biomarker concentrations. Potentiometry involves quantifying a sensor by measuring the open-circuit potential between the working and reference electrodes using a voltmeter. To interface with a high-impedance potentiometric sensor, a sensor is connected to a high-impedance voltage buffer to compensate for its high source impedance. For amperometry and voltammetry measurement, a potentiostat device is employed. This device controls the voltage difference between the working and reference electrodes while adjusting the current via a counter electrode in a three-electrode sensing setup. The concentration of a biomarker can be quantified by measuring the resulting current across the electrodes in response to the applied voltage difference. Two operational amplifiers, a control amplifier, and a transimpedance amplifier, are typically employed in a potentiostat circuit to control potentials and measure resulting current [4], or a dedicated analog front-end chip (e.g., AD5940, Analog Devices) can also execute potentiostatic techniques. The control amplifier provides bias voltage on the reference electrode and will adjust the current through the counter electrode to maintain the voltage difference between the working and reference electrodes. The transimpedance amplifier converts the amount of current across the counter and working electrode into a readable voltage difference, which can be processed by an analog-to-digital converter in a microcontroller. In optical-based sensing, such as colorimetric detection, sensing results can be visually observed by the naked eye or a camera. To quantify a color signal, a smartphone camera can serve as the sensing interface [118, 191]. Differences in color space, such as RGB and HSV, are analyzed in captured images to quantify the color signal [192].

The sensor interface must be coupled with a microcontroller unit and wireless communication module to process and transmit sensing data to a PC- or smartphone-based applications. The microcontroller and wireless communication module can be implemented using discrete components or a system-on-a-chip that integrates both functions into a single chip (e.g., CC2540, Texas Instruments). The microcontroller includes input/output peripherals (e.g., Digital-to-Analog and Analog-to-Digital converters) to manage the sensing interface and gather sensing data. The acquired sensing signals are then processed and stored using the built-in processor and memory. As wireless communication protocols, Bluetooth and near-field communication (NFC) are commonly used for wearable biosensing platforms, each with its own advantages and disadvantages. Bluetooth supports long-range communication up to 100 m [4], but a sufficient power source is required to operate long-term Bluetooth communication. Conversely, NFC operates over short distances of a few centimeters and requires a relatively large antenna. On the other hand, NFC can wirelessly harvest power through its antenna from an NFC reader, enabling battery-less operation of the sensing platform.

The power management circuit of monitoring electronics is crucial for ensuring reliable and efficient operation while maintaining a compact size and maximizing battery life. Generally, wearable microneedle platforms are powered via batteries. Power supplied from the battery is managed using a DC-DC converter and voltage regulator to provide the required operation voltage of each component. A battery charger with a charging coil can be included in the power management system for wireless charging of the battery [13]. Recent energy harvesting technologies, such as solar cells and TENG, have shown capabilities in energy capture from human motion and the environment. Through these technologies, harvesters can eliminate the need to charge batteries and extend the usability of microneedle sensing platforms [193]. Additional power converters, such as DC-DC boosters and bridge rectifying circuits, are utilized to integrate energy harvested from these sources effectively. These converters transform the irregular power output from energy harvesters into a stable DC output that meets the requirements necessary for monitoring electronics, ensuring continuous and sustainable operation over the long term.

Microneedle sensors have also been integrated with monitoring electronics to achieve continuous biomarker detection. For example, a touch-actuated glucose sensing microneedle was coupled with wireless electrochemical monitoring electronics and a smartphone application (Fig. 7B) [43]. The system consisted of an analog front-end (AFE) sensing module, a microcontroller unit (MCU), power modules, and reverse iontophoresis (RI) sweat extraction module (Fig. 7C). The power module supplied stable voltages to the MCU, RI sweat extraction module, and AFE sensing module using a voltage regulator and boost converter. The AFE chip executed amperometry measurements of the enzymatic glucose sensor. MCU and Bluetooth are integrated into a single system-on-chip, which transmits sensing signals to the smartphone-based application. Furthermore, a coin-sized personalized electronic reader for electrochemical transistors was designed with a customized analog MCU, a BLE unit, and other components (Fig. 7D) [179]. All components were meticulously assembled onto a coin-sized 15 mm × 15 mm printed circuit board. The miniaturized device footprint of the monitoring electronics offered user comfort and wearability. In addition to electrochemical-based sensing, a smartphone-based nucleic acid amplification platform using microneedle sensors was employed for fluorometric detection [191]. This platform enables the excitation of fluorescent dye using blue LED. The resulting fluorescent image was captured by a smartphone camera to detect DNA and RNA with 1 pg/mL sensitivity. These advancements illustrate the integration of microneedle sensors with monitoring electronics to pave the way for continuous biomarker detection and personalized health monitoring solutions.

## Applications

### Monitoring

Monitoring various biomarkers in ISF is a primary application of microneedle sensing platforms. The strong correlation in composition between ISF and blood makes microneedles a promising ISF-based diagnosis and monitoring tool because it eliminates the need for invasive blood sampling. Continuous glucose monitoring (CGM) is a representative application of a microneedle sensing platform [18]. Traditionally, diabetic patients rely on periodic fingerstick tests, which only offer snapshots of their glucose levels throughout the day. However, CGM using microneedles can provide real-time data, providing continuous information without the discomfort and inconvenience of frequent finger pricks. CGM devices have been successfully marketed. Several CGM systems, such as Freestyle Libre (Abbott), G7 (Dexcom), and Guardian sensor (Medtronic), are all commercially available. In addition to CGM, which uses a hypodermic needle, microneedle-based sensors for a range of analytes such as lactate (NCT04238611), methadone (NCT05998876), and levodopa (NCT04735627) have undergone clinical trials [92, 194, 195]. Several previous studies on microneedle sensors have demonstrated biomarker monitoring in humans [13, 21, 31, 136, 138, 160, 196]. While microneedle-based sensing devices haven’t reached commercialization yet, it is noteworthy that wearable sweat-based sensors such as the Gx Sweat Patch and Nix hydration biosensor have entered the market, demonstrating the demand for and the practicality of wearable biosensing devices.

**Fig. 8:**
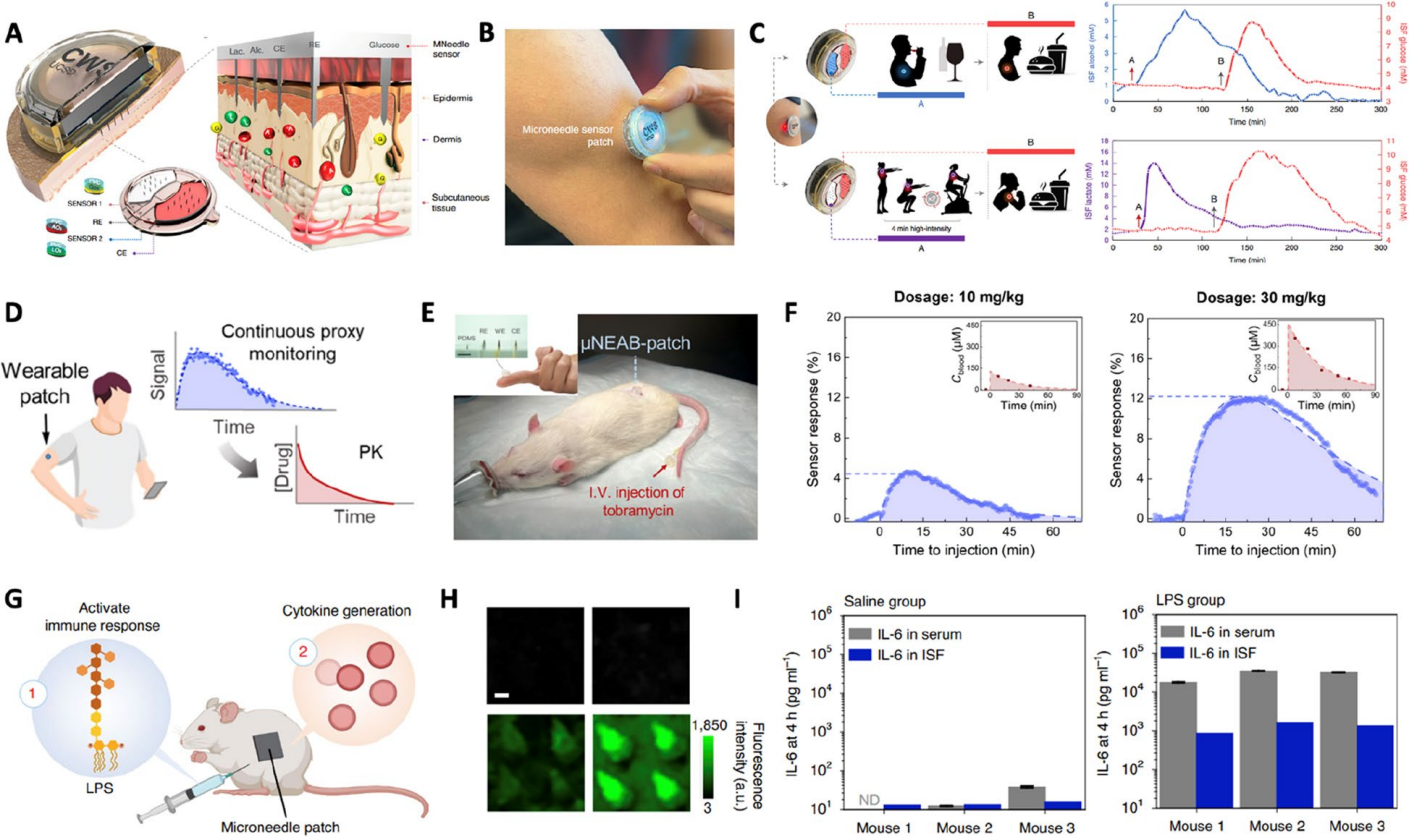
Monitoring applications. **A** Microneedle sensing patch for continuous and multiplexed detection. **B** Photograph of a microneedle sensor patch. **C** Continuous monitoring of alcohol, lactate, and glucose in ISF using an integrated microneedle sensing patch. Reproduced from [13] with permission.  Copyright 2022, Springer Nature Publishing. **D** Microneedle sensor for therapeutic drug monitoring. **E** Photography of a therapeutic drug monitoring microneedle patch attached on a rat. **F** Responses of microneedle sensor to different tobramycin doses. Reproduced from [34] with permission. Copyright 2022, American Association for the Advancement of Science. **G** Microneedle patch for monitoring immune response induced by lipopolysaccharide. **H** Fluorescent response of IL-6 sensing microneedle after lipopolysaccharide injection. **I** The concentration of IL-6 was measured by enzyme-linked immunosorbent assay (ELISA) (grey, serum) and the microneedle sensor (blue, ISF) after saline and lipopolysaccharide treatment. Reproduced from [38] with permission. Copyright 2021, Springer Nature Publishing.

Beyond single-analyte sensing like CGM, recent progress in microneedle sensing platforms has simultaneously addressed multiplexed detection of various analytes [13, 20, 177]. For example, a multiplexed microneedle sensor array comprising multiple enzyme-modified solid microneedles demonstrated simultaneous analyte monitoring of glucose, lactate, and alcohol on human subjects (Fig. 8A) [13]. The microneedle sensor patch is integrated with wireless electrochemical monitoring electronics which transmits sensing data to a smartphone application for real-time analyte analysis (Fig. 8B). The fully integrated microneedle sensing platform is capable of in vivo multiple-analyte measurements on freely moving humans during daily activities (Fig. 8C).

Continuous monitoring of drugs is also a promising application of the microneedle sensing platform [30–34]. For effective drug therapies, the drug concentration in the body should be administrated within its therapeutic window [197]. Conventional blood-based drug monitoring requires invasive sampling and may cause discomfort for patients. Since most drug molecules possess relatively small sizes, the diffusion of drugs through endothelial cell junctions and membranes can contribute to the correlation in drug concentration between blood and ISF [197]. Continuous and pain-free drug monitoring in ISF using a microneedle platform has great potential for future therapeutic drug monitoring. For example, a β‑Lactamase-immobilized microneedle sensor enables potentiometric measurement of penicillin concentration [31]. This microneedle sensor also demonstrated monitoring capabilities in human subjects [30]. In an animal study, a therapeutic drug monitoring microneedle patch was reported that continuously evaluates tobramycin and vancomycin levels in dermal ISF of mice (Fig. 8D) [34]. The microneedle sensor detects drug molecules through aptamer conformational changes when the aptamer binds to the target molecule. Through reading the increased peak height of square wave voltammetry under vancomycin and tobramycin, respectively, the drug level changes in mice were monitored over 1 h (Fig. 8E). The response of the sensor over time showed a proportional correlation to the dosage of the injected drug, demonstrating promising usability for precise dose control to meet a narrow therapeutic window (Fig. 8F).

In addition to small molecules, microneedle sensing platforms have quantified protein biomarkers in ISF [35–42]. Proteins play essential roles in biological functions within living organisms, making them valuable markers in disease research and diagnosis. For example, a nanogold-enhanced fluorophore-linked microneedle was developed which can selectively capture protein biomarkers, such as IL-6 cytokine, with an 800-fold lower LOD than conventional fluorophore-linked immunosorbent assay (FLISA) through plasmonic effect (Fig. 8G) [38]. The patch captured with analytes showed a brighter green light, which is intuitive and convenient for signal reading (Fig. 8H). Therefore, this microneedle sensing platform demonstrated minimally invasive monitoring of the inflammatory immune response by measuring a pro-inflammatory cytokine, interleukin 6 (IL-6). The responses of plasmonic fluor-enhanced microneedle sensors have a good correlation with protein biomarker levels in serum measured by conventional ELISA techniques (Fig. 8I). This microneedle sensing platform demonstrated the detection of protein biomarkers in mice.

### Integrated therapy

While the primary focus of the microneedle sensing platform lies in health monitoring and diagnosis, integrated microneedle-based therapy combines continuous monitoring capabilities with therapeutic interventions such as drug delivery [44–48, 198, 199] and physical stimulation [200]. The combination of sensing and treatment enables a closed-loop therapeutic system that controls treatment timing and dosage based on the real-time recordings of biomarker levels using an in-situ microneedle sensor. With significant advancements in both microneedle-based sensing and therapeutic applications, this integrated approach holds great promise for precise and personalized medicine.

**Fig. 9:**
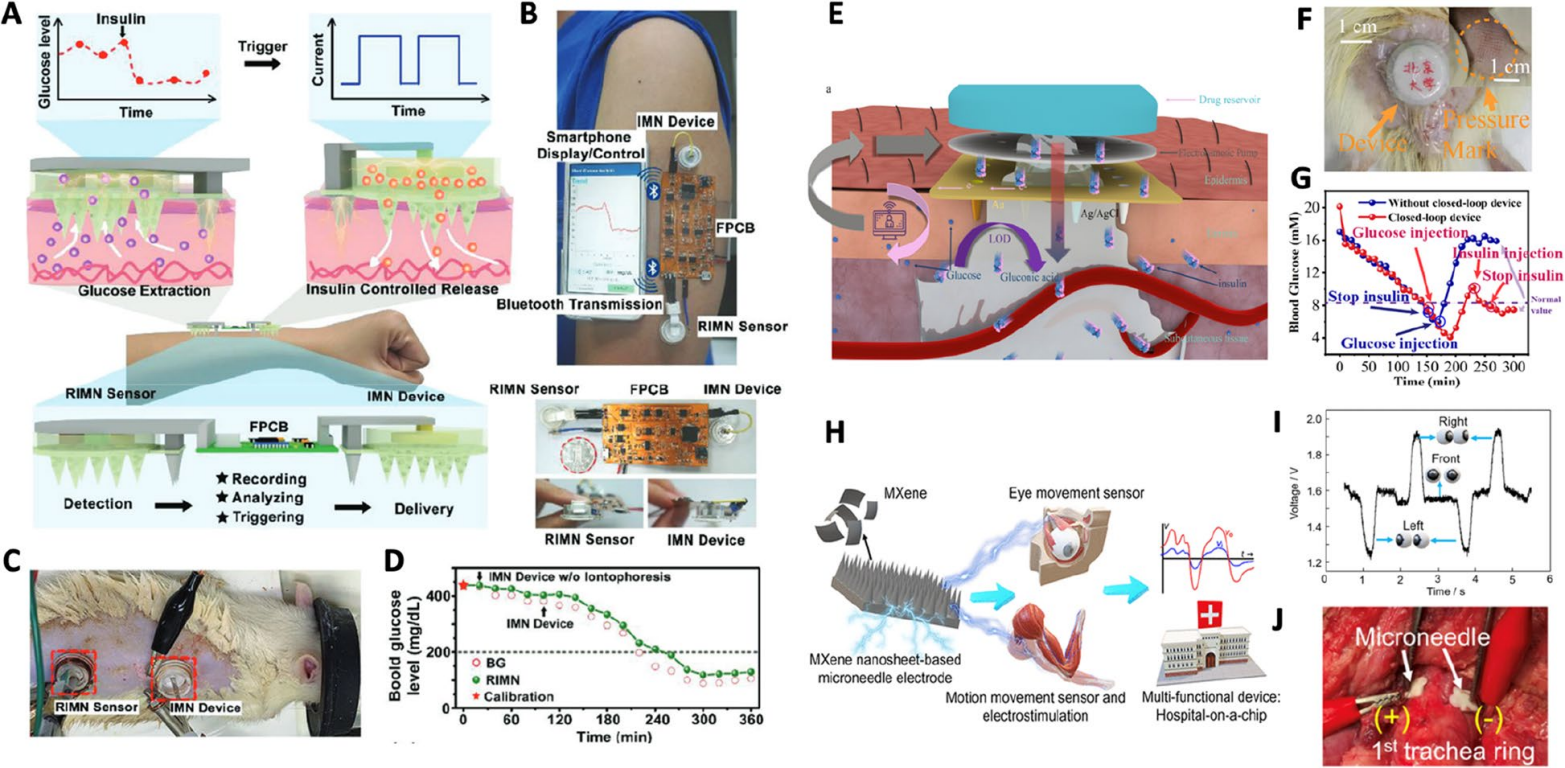
Integrated therapy. **A**, **B** Schematic (**A**) and photograph (**B**) of integrated microneedle platform for real-time glucose monitoring and in-situ insulin treatment. **C** Photograph of the microneedle glucose sensor and insulin-releasing microneedle on a diabetic rat. **D** Controlled insulin delivery on a diabetic rat using real-time measurement of glucose level. Reproduced from [44] with permission.  Copyright 2021, Wiley–VCH. E , F Schematic (**E**) and photograph (**F**) of the miniaturized, closed-looped microneedle patch for continuous glucose monitoring and insulin delivery for diabetic management. **G** The effect of closed-loop insulin treatment on a diabetic rat using the integrated microneedle patch. Reproduced from [46] with permission. Copyright 2022, American Chemical Society. **H** Integrated microneedle platform for biosensing and electrostimulation. I Measurement of voltage difference generated by the eye movement. **J** Electrostimulation on the muscles of the swine using microneedles. Reproduced from [200] with permission. Copyright 2021, American Chemical Society.

For instance, an integrated microneedle-based sensing and therapeutic platform proves beneficial in managing diabetes. Diabetes patients struggle to maintain normal blood glucose levels due to inadequate insulin production by the pancreas or insulin resistance. Managing diabetes typically involves regular blood glucose monitoring through invasive fingerstick tests with a glucometer. In cases of Type 1 diabetes, where the pancreas cannot produce sufficient insulin, patients require insulin injections. Microneedle-based systems offer an automated approach to controlling blood glucose levels by combining glucose sensing with insulin delivery capabilities, thereby enhancing patient convenience [44–47]. A fully integrated wearable microneedle platform has been developed for closed-loop diabetes treatment within a rat model (Fig. 9A). This platform incorporates a mesoporous microneedle sensor for ISF glucose monitoring, coupled with reverse iontophoretic glucose extraction, iontophoretic insulin delivery microneedles, and flexible printed circuit boards (FPCB) as the monitoring and control electronics (Fig. 9B). The system continuously monitors ISF glucose levels using the in-situ microneedle sensor and triggers iontophoretic insulin release when glucose levels exceed hyperglycemic thresholds. The integrated microneedle platform demonstrated its capability in closed-loop diabetes management in a diabetic rat model (Fig. 9C and D). Similarly, a miniaturized closed-loop microneedle patch was developed for diabetes management (Fig. 9E). This patch comprises biodegradable hollow microneedles divided into glucose sensing and insulin delivering sections. The outer parts of the microneedles are functionalized with the glucose oxidase enzyme for glucose monitoring, while the inner fluidic channels of the microneedles are coupled with a flexible electroosmotic pump for insulin delivery in response to monitored glucose levels. The microneedle array and controller are integrated into a compact patch with a 2.5 cm diameter, significantly improving the platform's wearability (Fig. 9F). This integrated microneedle platform effectively maintains glucose levels within a normal range through closed-loop insulin treatment in diabetic rat models (Fig. 9G).

In addition to diabetes management, microneedle sensing and therapeutic platform can be utilized for wound healing and monitoring [198, 199]. Metal–organic frameworks hydrogel microneedle contains curcumin to promote wound healing through antimicrobial and anti-inflammatory properties [199]. This multifunctional microneedle sensing patch enables the monitoring of the wound healing process in-vivo by the change in pH using a fluorescent agent. The wound management capability was demonstrated in a mice model.

Although the primary focus of integrated therapy is feedback-controlled drug delivery, such as insulin injection, electrostimulation can also be integrated with a microneedle sensing platform. A MXene nanosheet-based microneedle system integrated a motion movement sensor with electrostimulation treatment (Fig. 9H) [200]. This microneedle platform can detect tiny electrical potential differences caused by muscle contraction, such as arm and eye movement of human subjects (Fig. 9I). The MXene nanosheet-based microneedle demonstrated electrostimulation treatment in a swine model, which can potentially offer closed-loop electrostimulation therapy (Fig. 9J).

## Challenges and outlook

The advancement of microneedle sensing platforms marks a significant leap forward in the field of personalized healthcare and wearable biosensing devices. This review discussed diverse aspects of microneedle sensing technology while highlighting its potential as a daily health monitoring and diagnosis platform. Integrating microneedles with variable sensing mechanisms and miniaturized monitoring electronics offers minimally invasive and continuous detection methods for various biomarkers in ISF. With the significant advances in microneedle sensing technology, wearable ISF analyzing devices can potentially replace blood-based gold standard diagnostics in the future.

Despite the promise of microneedle sensing technology, several challenges must be addressed to bring microneedle sensing platforms to clinical and translational applications. First, Although ISF and blood are expected to have similar compositions, many analytes' diffusion processes and exchange mechanisms are still not fully understood [3, 17]. Extensive clinical studies of ISF-based diagnostics are needed to provide precise diagnoses of specific diseases. Further investigation is required into the detailed correlation between ISF and blood and clinical data on ISF-based diagnostics for particular diseases and abnormal health states using microneedle sensors.

For clinical trials, ensuring the biosafety of microneedle sensors is crucial. Fortunately, the risk of infection associated with microneedles has shown to be low despite their penetration of the outer skin layer [195, 201]. To guarantee long-term safety, it is essential to thoroughly investigate the biocompatibility of the materials used in the microneedle structure and sensing layer. Additionally, a robust sterilization protocol for microneedles must be established. Various sterilization methods can be utilized, such as steam autoclave, gamma irradiation, and dry heat [202]. However, it is important to note that the sterilization process may degrade sensing performance [119]. For instance, an aptamer-based sensing layer may be compromised after sterilization, and the degree of performance degradation can vary depending on the sterilization technique used [203]. Therefore, optimizing sterilization methods is necessary to minimize any negative impact on the sensor's performance.

In addition, establishing in vivo calibration protocols for microneedle-based sensors is crucial to acquiring accurate biomarker concentrations in ISF. Microneedle sensors may face biofouling and degradation inside the skin, necessitating regular in-situ recalibration during long-term use. Currently, microneedle sensor responses are often compared with blood concentrations, which may validate qualitative trends but not provide accurate quantitative information in ISF. Occasional sampling of ISF combined with off-device analysis can be used alongside continuous monitoring to calibrate and correct the sensing signals from the microneedle sensor embedded in the skin. To ensure consistency, the method of ISF collection should be standardized to minimize deviations caused by different sampling techniques [172].

For daily use, reproducible insertion of the microneedle sensor is important to ensure the desired sensing performance. Application force and velocity influence the depth of microneedle insertion. While thumb pressure is commonly used to apply microneedles to the skin, a dedicated applicator can provide more controlled pressure and velocity. This allows for more reproducible and efficient insertion of the microneedles [184, 204]. Furthermore, a well-designed microneedle with a sharp tip can improve the ease and consistency of insertion.

On the fabrication side, while various methods and materials have been developed, there remains a demand for manufacturing methods with high scalability, resolution, and design flexibility. Scalable fabrication methods often lack resolution and design flexibility, compromising the sharpness and microstructure of microneedle designs. This limitation can deteriorate sensing performance and increase user discomfort. Additionally, the limited volume of extracted ISF and the complex shape of microneedle structures make it challenging to meet desirable sensing performances such as temporal resolution, sensing range, and sensitivity.

Lastly, miniaturized, integrated, and self-powered monitoring electronics with conformable form factors can significantly improve the wearability of microneedle sensing platforms in daily activities. Artificial intelligence-empowered multimodal data analytics can also further enhance the precision of diagnosis when applied to microneedle sensing platforms [205–207]. Overall, with the recent advancements covered and limitations addressed, the possibility of continuous, sensitive, and mass-producible development of microneedle sensors for dermal ISF monitoring draws closer to being a reality for personalized healthcare.
